# Tumour epithelial vimentin expression and outcome of pancreatic ductal adenocarcinomas

**DOI:** 10.1038/bjc.2011.93

**Published:** 2011-03-29

**Authors:** A Handra-Luca, S-M Hong, K Walter, C Wolfgang, R Hruban, M Goggins

**Affiliations:** 1 Department of Pathology, The Sol Goldman Pancreatic Cancer Research Center, The Johns Hopkins Medical Institutions, Baltimore, MD, USA; 2 The UFR SMBH Universite Paris 13/Nord Medecine APHP, Paris, France; 3 Department of Surgery, The Sol Goldman Pancreatic Cancer Research Center, The Johns Hopkins Medical Institutions, Baltimore, MD, USA; 4 Department of Medicine, The Sol Goldman Pancreatic Cancer Research Center, The Johns Hopkins Medical Institutions, Baltimore, MD, USA; 5 Department of Oncology, The Sol Goldman Pancreatic Cancer Research Center, The Johns Hopkins Medical Institutions, Baltimore, MD, USA

**Keywords:** pancreas, adenocarcinoma, prognosis, vimentin

## Abstract

**Purpose:**

Tumour epithelial vimentin expression is a marker of mesenchymal differentiation and may be a useful marker of carcinomas with more aggressive behaviour. The aim of this study was to determine the extent and prognostic significance of vimentin expression in pancreatic ductal adenocarcinomas.

**Methods:**

Vimentin expression was detected by immunohistochemistry on tissue microarrays of surgically resected pancreatic ductal adenocarcinomas from 387 patients. The percentage of vimentin-immunolabelled neoplastic cells was correlated with outcome and with clinico–pathological factors using the Kaplan–Meier method and Cox multivariate survival models.

**Results:**

In all, 45% of primary pancreatic adenocarcinomas contained neoplastic cells that expressed vimentin, and in 27.5% of the cancers >10% of cells expressed vimentin. Vimentin expression was correlated with poor histological differentiation. By both uni- and multivariate survival analysis, neoplastic vimentin expression (*P*<0.01, HR 1.52, 95% confidence interval 1.14–2.04) was an indicator of a shorter postsurgical survival independent of other clinico–pathological variables.

**Conclusion:**

The presence of vimentin-expressing tumour epithelial cells in surgically resected pancreatic adenocarcinomas independently predicted a shorter postsurgical survival.

Pancreatic adenocarcinoma is the fourth leading cause of cancer death in the United States. Although there have been numerous advances in our understanding of pancreatic cancer development and progression in recent years, therapies for pancreatic cancer generally still provide only modest benefit. Surgical resection is currently the most effective treatment but is undertaken in only ∼15–20% of patients highlighting the need for early detection strategies. Even among patients with resectable pancreatic cancer survival is poor, with <20% of patients alive at 5 years Among patients undergoing pancreatic resection with curative intent, the main prognostic factors are histological grade, resection margin status, tumour size, location and lymph node metastasis (Winter *et al*, 2006; Corsini *et al*, 2008; Herman *et al*, 2008; Goggins, 2011 (in press); Vincent *et al*, 2011 (in press)).

Although very useful, these pathological risk factors do not sufficiently predict outcome and most of these prognostic factors reflect tumour stage rather than tumour epithelial biology. Multiple studies have attempted to identify markers that may assist in predicting outcome after pancreatic cancer resection and that may also provide clues as to biological mechanisms that contribute to pancreatic cancer aggressiveness. For example, mutations or loss of expression of tumour-suppressor proteins expressed by tumour epithelial cells such as Smad4 (Tascilar *et al*, 2001; Biankin *et al*, 2002; Blackford *et al*, 2009; Iacobuzio-Donahue *et al*, 2009), or patterns of stromal fibroblast-expressed proteins such as Sparc have been shown to predict outcome (Sato *et al*, 2003; Infante *et al*, 2007).

Vimentin is expressed by normal mesenchymal tissue and is considered a marker of mesenchymal differentiation (Leader *et al*, 1987). Vimentin is an intermediate-sized filament polypeptide, which along with desmin, keratin, glial acidic protein and neurofilament intermediate filaments are distinguished by their chemical characteristics, immunological specificities and cell-type distribution (Dellagi *et al*, 1983; Lazarides *et al*, 1982). Epithelial neoplasms can occasionally express both cytokeratin and vimentin, features often associated with the acquisition of mesenchymal histologic features. There is evidence that carcinomas with markers of mesenchymal differentiation have different biological and clinical behaviour (Domagala *et al*, 1990a,1990b; Medeiros *et al*, 1988; Liu *et al*, 2010). Pancreatic cancer vimentin expression patterns have been investigated in small series. Schussler *et al* (1992) reported a lack of expression in pancreatic cancers, while Nakajima *et al* (2004) observed sparse cell labelling in 30 primary pancreatic adenocarcinomas with prominent expression in 15 liver metastases. Similarly, vimentin labelling was more pronounced in widely metastatic pancreatic cancers as compared with locally destructive ones (Naito and Iacobuzio-Donahue, 2010). Khoury *et al* found that vimentin expression in 34 pancreatic cancers correlated with poor survival (Javle *et al*, 2007). Interestingly, although mesenchymal differentiation is associated with reduced E-cadherin expression *in vitro*, in liver metastasis of pancreatic ductal adenocarcinomas Nakajima *et al* (2004) found a correlation between vimentin expression and N-cadherin but not E-cadherin expression. More recent reports suggest that, *in vitro,* most pancreatic cancers with well-defined glandular differentiation do not show the downregulation of E-cadherin, although focal loss of E-cadherin expression is observed in some primary pancreatic cancers (Li *et al*, 2010; Hong *et al*, 2011 (in press)) and undifferentiated pancreatic cancers often display complete loss of E-cadherin expression (Winter *et al*, 2008).

In this study, we investigated vimentin expression in a large series of pancreatic ductal adenocarcinomas treated by surgery and assessed its relationship to survival.

## Patients and Methods

### Patients and samples

Patients having conventional pancreatic ductal adenocarcinoma (Kloppel *et al*, 2000), treated by surgical resection were retrieved from the database of the Pathology Department, of the Johns Hopkins Medical Institutions, Baltimore, MD, USA. Between 1998 and 2006, 387 patients had available neoplastic tissue blocks to construct tissue microarrays.

This study was designed and performed according to current recommendations for tumour markers (McShane *et al*, 2005). The patients were analysed for clinical and pathological data according to standard criteria (Kloppel *et al*, 2000; AJCC, 2010). The clinical data analysed included: age, gender, date of surgery, type of surgery, stage, date of death or last consultation for the postsurgical survival. As information on adjuvant therapy was not complete for some patients who received their postoperative therapy at other centres, postoperative therapy was not included in the analysis of the prognostic significance of vimentin. The analysed pathological data were: tumour size, histological differentiation, vascular invasion, perineural invasion, pathological T TMN stage, lymph node metastases and N TMN stage as well as for distant metastases (M TNM stage). As their outcome is significantly different patients were excluded if they had a peri-operative death (occurring within the first 30 days postoperatively, 4 patients) or with follow-up of <30 days (28 patients), a distal splenopancreatectomy (1 patients) or if they received neoadjuvant radiotherapy (2 patients).

### Immunohistochemistry

Vimentin protein expression was assessed in the tumour epithelial tissues by immunohistochemistry. Tissue microarrays were constructed from representative areas of neoplastic epithelial cells and normal pancreas as previously described (Infante *et al*, 2007; Matsubayashi *et al*, 2007; Walter *et al*, 2010). Each patient's tissue was represented on the tissue microarrays by two cores of pancreatic cancer and two cores of non-neoplastic pancreas.

Immunohistochemistry was performed using an antibody to vimentin protein (Dakocytomation, Carpinteria, CA, USA, clone V9, dilution 1 : 200). Protein expression in the cytoplasm or peri-membrane and membrane in epithelial malignant cells setting was assessed by an experienced pancreatic pathologist (AHL) at an Olympus BX51 microscope (Olympus, Center Valley, PA, USA). The percentage of labelled tumour epithelial cells was determined. Spindle-shaped isolated cells with bland, regular nucleus were considered as stromal fibroblasts. Representative neoplastic zones were photographed and were included in the figures (Photo Olympus DP20). For 14 patients, there were no data on vimentin neoplastic cell expression because of tissue loss during the immunolabelling protocol. For each neoplasm, the core with the highest expression of vimentin was taken into consideration for subsequent statistical analysis. The percentage of labelled neoplastic epithelial cells ranged from 0 to 95%.

### Statistical analysis

For the statistical analysis, the variables were considered as categorical. For continuous variables such as tumour size or lymph node metastases, the cutoff for classifying the tumours was the median. We also determined the median percentage of vimentin-expressing neoplastic cells, which was 1% and used this as a cutoff for classifying cancers. We also evaluated other cutoffs (0, 10, 20, 30, 40, 50, 60, 70, 80 and 90%). According to the distribution of the percentage of vimentin-expressing neoplastic cells in this series of tumours, the cutoff of 10% was accepted as most predictive. The relationships between clinico–morphological variables and the expression of vimentin by neoplastic cells were analysed by using the Fisher's or *χ*^2^-tests (Medcalc v11.1.1 software, Medcalc, Mariakerke, Belgium). For univariate survival analysis we used the Kaplan–Meier method, the survival curves being compared by the log-rank test. For multivariate survival analysis we used the Cox method. Variables found on univariate analysis to be related to postsurgical survival with a *P*-value of <0.05 were included in Cox models. Colinearity (redundancy) of variables being significantly correlated on Fisher's or *χ*^2^-tests was tested (neoplastic differentiation and vimentin expression, microscopic vascular expression and lymph node metastases, tumour size and type of surgery). Patients with unavailable data were included in the analysis as ‘unknown’. For all the statistical tests and methods, a *P*-value of <0.05 was used for defining statistical significance (Christensen, 1987; Chen and Wang, 1991; Hosmer and Lemeshow, 2000).

## Results

### Patients’ and tumour characteristics

The patients’ demographics are listed in Table 1. The median follow-up was 14.4 months (range, 1.1–101.95 months). During the study period, 246 patients died (excluding patients with perioperative death) and the median survival was 14.4 months and the time to death was 12.90 months (range, 1.21–59.24 months). The 3-year postsurgical survival was 19.32% and the 5-year survival 8.0%.

Median tumour size was 30 mm (range, 10–120 mm). Tumour size was significantly higher in those patients having total pancreatectomy surgery as compared with those treated by pancreaticoduodenectomy (*P*=0.04). Lymph node metastases were observed in 85.7% of the patients with the median number of metastatic lymph nodes being 3 (range, 1–25). The presence of lymph node metastases was correlated to vascular and perineural invasion (*P*<0.01 and *P*=0.05, respectively), whereas presence of >3 lymph node metastases was correlated to vascular invasion and increased neoplastic size (*P*<0.01 and *P*=0.02, respectively). Three patients showed distant metastasis (pericaval, mesocolon and subcostal skin metastases, respectively).

### Vimentin expression by pancreatic cancer cells

**Figure 1:**
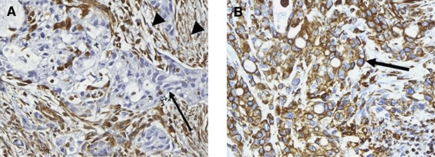
Vimentin protein expression in primary pancreatic adenocarcinoma cells: the figure on the left (**A**) shows minimal vimentin expression in a few pancreatic cancer cells (arrow), vimentin also being expressed in stromal fibroblasts (considered as internal control, arrowheads). On the right side (**B**) there is pancreatic adenocarcinoma showing uniform cytoplasmic and perimembrane pattern of vimentin expression (arrow) (original magnification × 30).

**Figure 2:**
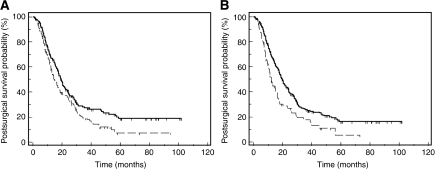
Kaplan–Meier curves for postsurgical survival according to tumour epithelial vimentin expression when considering the cutoff of 1% (**A**) and that of 10% (**B**). The continuous line indicates the patients with high vimentin (>1 or >10%) expression whereas the discontinuous lines those patients with low vimentin expression.

Vimentin expression by the neoplastic cells was observed in 154 (45%) pancreatic ductal adenocarcinomas (Figure 1), and included a wide variation in the extent of cancer cell expression varying from 1 to 95%, with the median percentage of vimentin-labelled cancer cells being 1%. In 94 pancreatic cancers (27.5%), vimentin was expressed in >10% of neoplastic cells. There was not a specific labelling pattern, vimentin being expressed by cells with varying degrees of cytonuclear atypia, with or without intracellular mucus, forming neoplastic glands or masses or disposed isolated within the stroma. When analysed with regard to the morphological features of the neoplasm, the expression of vimentin by neoplastic cells was significantly correlated only with poor histological differentiation (using the vimentin cutoff of 1%, 76 of 148 poorly differentiated cancers compared with 78 of 194 well and moderately differentiated cancers; using the vimentin cutoff of 10%, 53 of 143 poorly differentiated neoplastic cells *vs* 41 of 194 well and moderately differentiated cancers) (*P*=0.05 and *P*<0.01 for vimentin cutoffs 1% and 10%, respectively). There was no statistical difference in neoplastic cell vimentin expression between early-stage (stages T1 or T2 TNM) and advanced tumours (TNM stages T3 or T4). By univariate survival analysis (Table 1), patients’ outcome was correlated with poor histological differentiation, positive tumour resection margins, the presence of lymph node metastases, increased tumour size and tumour epithelial vimentin expression. The statistical significance of the relationship between tumour epithelial vimentin expression and a shorter postsurgical survival was more important when considering the cutoff of 10% (*P*<0.01, median postsurgical survivals 12.49 *vs* 19.72 months) (Figure 2) than for the cutoff of 1% (*P*=0.02, median postsurgical survivals 15.05 *vs* 19.52 months). By multivariate survival analysis (Table 2), tumour epithelial vimentin expression was significantly related to a shorter postsurgical survival, independently of the degree of histological differentiation, surgical margin status, tumour size (>30 mm) and lymph node metastases. In this model, high (>10%) tumour epithelial vimentin expression was a more powerful predictor of outcome than the N TNM stage, the type of surgery, and tumour size, the risk of death being 1.53.

High tumour epithelial vimentin expression correlated with 3- and 5-year survival (*P*<0.01 for both comparisons) as well as tumour size (*P*=0.03 and *P*=0.05), N TNM stage (*P*<0.01 and *P*=0.03), differentiation (*P*<0.01 for both comparisons), surgery type (*P*=0.03 and *P*=0.05), and margin status (*P*<0.01 and *P*=0.02).

**Figure 3:**
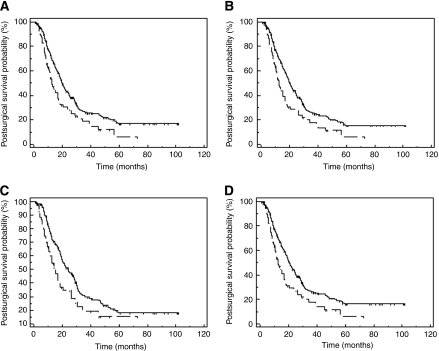
Kaplan–Meier curves for postsurgical survival according to tumour epithelial vimentin expression when considering varied groups of patients: groups of patients treated by pancreaticoduodenectomy (**A**), patients with pathological T3 TNM stage (**B**), patients with tumour-free surgical resection (**C**) as well as patients with stage 2 disease (**D**). The continuous line indicates the patients with high vimentin (>10%) expression whereas the discontinuous lines those patients with low vimentin expression.

The association of positive vimentin expression with a shorter survival remained among the main subgroups of patients studied including those treated by pancreaticoduodenectomy, with T3 stage cancers, those patients with lymph node metastasis (N1 TNM stage) and those patients having margin negative, stage 2 or stage 2B disease or in those patients having cancers with moderate or poor differentiation (Table 3) (Figure 3).

Similarly, in the group of patients having cancers with high vimentin expression (>10% of cancer cells expressing), margin-positive surgical resection (*P*=0.04) and tumour size (*P*=0.04) were also predictors of a shorter postsurgical survival.

## Discussion

Our analysis of a large series of pancreatic adenocarcinomas treated by surgical resection indicated that tumour epithelial vimentin expression is an indicator of adverse outcome both on univariate and multivariate survival analysis, independently of classical tumour characteristics such as differentiation, tumour size, resection margin status and type of surgical treatment.

The most powerful predictors of a shorter postsurgical survival in our series were positive margin status and poor histological differentiation, in agreement with the results of Herman *et al* (2008), and Winter *et al* (2006), in previous studies from our institution. We also found that lymph node metastasis predicted a shorter postsurgical survival, consistent with already reported results (Winter *et al*, 2006; Infante *et al*, 2007; Chang *et al*, 2009).

Vimentin expression by neoplastic cells was observed in 45% of the pancreatic adenocarcinomas and an expression level of >10% was noted in 27.5% of the pancreatic cancers. We found this 10% cutoff to be more predictive of outcome than other cutoffs of percentage expression or the absolute cutoff (of presence *vs* absence of neoplastic cell expression). Although the expression concerned a limited percentage of neoplastic cells in some tumours, vimentin was significantly related to postsurgical survival. The association of vimentin expression to a shorter survival had a greater prognostic significance than that of N TNM stage, tumour size, and of almost as much magnitude as other prognostic factors such as differentiation and surgical margin (Winter *et al*, 2006; Herman *et al*, 2008; Chang *et al*, 2009).

The underlying biological mechanisms that might explain the relationship between vimentin and survival are not known. As has been previously reported for breast and prostatic adenocarcinomas (Domagala *et al*, 1990a,1990b; Heatley *et al*, 1993; Zhao *et al*, 2008), we found a correlation between vimentin expression and histological differentiation. Previous studies have found associations between vimentin expression and cancer cell morphology in tumour xenografts (Neureiter *et al*, 2005). Vimentin expression in neoplastic epithelial cells might reflect a reorganisation of cytoplasmic intermediate filaments, which could be related to poor differentiation. However, poor differentiation is more complex than cytoplasmic changes since it involves also nuclear changes. Although vimentin expression is associated with poor differentiation in cancers, in our series vimentin expression is still an independent predictor of outcome after accounting for the degree of differentiation suggesting vimentin expression is a marker for more than differentiation. Whether or not vimentin expression directly affects the aggressiveness of pancreatic cancer cells because of its functional effects as an intermediate filament or whether it is merely a marker of a more aggressive cancer cell is not known. Studies of peripheral blood mononuclear cells as well as of human breast, colon and prostate cancer cell lines suggest a cell-type-specific role for vimentin in cell adhesion, motility and invasiveness (Nieminen *et al*, 2006; Ivaska *et al*, 2007; McInroy and Määttä, 2007; Zhao *et al*, 2008). Vimentin's functions are influenced by cell signalling pathways such as AKT and STK33 that result in vimentin phosphorylation (Ivaska *et al*, 2007; Brauksiepe *et al*, 2008; Zhu *et al*, 2011). Vimentin phosphorylation contributes to disassembly of vimentin polymers diminishing its function as an intermediate filament and influencing its protein–protein interactions. Vimentin also undergoes proteolysis by caspases when cells receive pro-apoptotic stimuli (Lahat *et al*, 2010). Recently vimentin has evolved as a marker of ‘epithelial–mesenchymal transdifferentiation’ and many molecular alterations have been implicated in this process including Notch, miR-200 and others (Klymkowsky and Savagner, 2009). Cancer cell vimentin expression has also been found to influence treatment response *in vitro*. For example, gemcitabine-resistant pancreatic cancer cells display increased vimentin expression (Traub *et al*, 1985), and vimentin expression increased in Panc-1 cell lines when treated by TGFbeta (Nakajima *et al*, 2004). Vimentin has been investigated as a therapeutic target. A natural compound, withaferin-A-induced vimentin degradation and slowed the growth of sarcomas and had proapoptotic effects in sarcoma and carcinoma cell lines expressing vimentin (Lahat *et al*, 2010).

In recent years, vimentin expression has been commonly used as a marker of mesenchymal phenotypes but this intermediate filament has complex functions including its affinity to single-stranded and supercoiled DNA and its strong tendency to interact with different chromatin constituents including histones (Traub *et al*, 1985). However, mice lacking vimentin do not have altered phenotypes so the functional significance of vimentin expression is not well understood (Colucci-Guyon *et al*, 1994).

As vimentin is highly expressed in stromal fibroblasts it is not likely to be useful as a marker for differentiating pancreatic cancers from pancreatitis, but interestingly autoantibodies to this protein have been recently reported (Hong *et al*, 2006). Pancreatic neoplastic tissues showed a three-fold higher expression of vimentin than neoplasms of other origins (lung, colon and ovary), and had higher levels of an isoform with demonstrable immunogenicity (Hong *et al*, 2006). More promising as a marker of neoplasia is vimentin promoter methylation, which is being evaluated as a candidate marker of colorectal carcinomas (Chen *et al*, 2005; Zou *et al*, 2007; Shirahata *et al*, 2009).

There are a number of limitations to our study that should be acknowledged. First, our study is a single-centre study, and confirmation of our findings in other series is warranted. Second, our prognostic model did not include postoperative therapy in our model as unlike surgical therapy, postoperative therapy is not uniform in our study population. Although our cases were not selected with regard to their postoperative course, it is possible that the association between tumour epithelial vimentin expression and outcome of pancreatic cancer could be influenced by differences in postoperative chemoradiotherapy.

In conclusion, the results of our study suggest that *de novo* tumour epithelial expression of vimentin in pancreatic ductal adenocarcinoma is an independent predictor of adverse postsurgical outcome.

## Figures and Tables

**Table 1:** Clinico–pathological characteristics of the patients with pancreatic ductal adenocarcinomas treated by surgical resection

	**Number of patients *n*=356^a^**	**Median postsurgical survival (months)**	**Log-rank *P***
*Gender*			0.83
Women	160	16.76	
Men	196	18.08	
			
*Age (years)*			
Range	32–90		
Median	66.66		
			
*Outcome*			
Follow-up	14.4		
Range, months	1.08–101.95		
Median, months		14.4	
Dead	246		
Alive	110		
			
*T Stage*			0.12
pT1	4	—	
pT2	7	17.44	
pT3	336	17.98	
pT4	9	10.29	
			
*Differentiation*			<0.01
Well, moderate	199	21.89	
Poor	157	13.01	
			
*Vascular invasion*			0.02
Absent	170	20.38	
Present	176	15.32	
Indeterminate	10	12.82	
			
*Surgery type*			0.04
Pancreaticoduodenectomy	335	17.98	
Total pancreatectomy	21	10.75	
			
*Tumour size*			0.02
Range, median			
30 mm	217	19.59	
>30 mm	139	14.66	
			
*Surgical margins*			<0.01
Non-tumoral	238	20.31	
Tumoral	118	14	
			
*Lymph node metastasis*			
*N TNM stage*			0.01
N0	51	26.4	
N1	305	16.63	

Abbreviations: *n*=number of patients; *N*=node; T=tumour; p=pathology; TNM=tumour node metastasis classification.

a Patients with perioperative death (within the 30 days after surgery), were not included for the statistical analysis.

**Table 2:** Cox model including as variables tumour and surgical resection parameters

	***P*-value**	**Hazard ratio**	**95% confidence interval**
Differentiation	<0.01	1.54	1.20 to 1.96
Surgical margin status	<0.01	1.62	1.24 to 2.12
Tumour vimentin expression	<0.01	1.53	1.14 to 2.05
Type of surgery	0.03	1.81	1.07 to 3.08
N TNM stage	0.05	1.48	1.00 to 2.18
Tumour size, >3 mm	0.14	1.22	0.94 to 1.59

Abbreviations: *N*=node; TNM=tumour node metastasis classification.

**Table 3:** Univariate survival analysis for vimentin expression in pancreatic ductal adenocarcinomas

	**Median postsurgical survival (months)**	**Log-rank *P-*value**
*Patients treated by pancreaticoduodenectomy (*n*=322)*		<0.01
Low tumour vimentin	19.72	
High tumour vimentin	12.82	
		
*T3 TNM stage patients (*N*=322)*		<0.01
Low tumour vimentin	20.35	
High tumour vimentin	12.49	
		
*N1 TNM stage patients (*N*=293)*		<0.01
Low tumour vimentin	19.29	
High tumour vimentin	12.09	
		
*Stage 2 patients (AJCC 7) (*N*=328)*		<0.01
Low tumour vimentin	20.38	
High tumour vimentin	12.49	
		
*Stage 2B patients (AJCC 7) (*N*=279)*		<0.01
Low tumour vimentin	20.35	
High tumour vimentin	12.29	
		
*Patients with margin negative surgical resections (*N*=230)*		0.02
Low tumour vimentin	14.95	
High tumour vimentin	10.25	
		
*Patients having tumours with moderate or poor histological differentiation (*N*=332)*		<0.01
Low tumour vimentin	19.46	
High tumour vimentin	12.49	

Abbreviations: AJCC=American Joint Committee on Cancer; *n*=number of patients.

## References

[bib1] AJCC (2010) American Joint Committee on Cancer: AJCC Cancer Staging Manual. 7th edn. In: Edge SB, Byrd DR, Compton CC, Fritz AG, Greene FL, Trotti A (eds) Springer: New York

[bib2] Biankin AV, Morey AL, Lee CS, Kench JG, Biankin SA, Hook HC, Head DR, Hugh TB, Sutherland RL, Henshall SM (2002) DPC4/Smad4 expression and outcome in pancreatic ductal adenocarcinoma. J Clin Oncol 20: 4531–4542 12454109 10.1200/JCO.2002.12.063

[bib3] Blackford A, Serrano OK, Wolfgang CL, Parmigiani G, Jones S, Zhang X, Parsons DW, Lin JC, Leary RJ, Eshleman JR, Goggins M, Jaffee EM, Iacobuzio-Donahue CA, Maitra A, Cameron JL, Olino K, Schulick R, Winter J, Herman JM, Laheru D, Klein AP, Vogelstein B, Kinzler KW, Velculescu VE, Hruban RH (2009) SMAD4 gene mutations are associated with poor prognosis in pancreatic cancer. Clin Cancer Res 15: 4674–4679 19584151 10.1158/1078-0432.CCR-09-0227 PMC2819274

[bib4] Brauksiepe B, Mujica AO, Herrmann H, Schmidt ER (2008) The serine/threonine kinase Stk33 exhibits autophosphorylation and phosphorylates the intermediate filament protein vimentin. BMC Biochem 9: 25 18811945 10.1186/1471-2091-9-25 PMC2567967

[bib5] Chang DK, Johns AL, Merrett ND, Gill AJ, Colvin EK, Scarlett CJ, Nguyen NQ, Leong RW, Cosman PH, Kelly MI, Sutherland RL, Henshall SM, Kench JG, Biankin AV (2009) Margin clearance and outcome in resected pancreatic cancer. J Clin Oncol 27: 2855–2862 19398572 10.1200/JCO.2008.20.5104

[bib6] Chen CH, Wang PC (1991) Diagnostic plots in Cox's regression model. Biometrics 47: 841–850 1742442

[bib7] Chen WD, Han ZJ, Skoletzky J, Olson J, Sah J, Myeroff L, Platzer P, Lu S, Dawson D, Willis J, Pretlow TP, Lutterbaugh J, Kasturi L, Willson JK, Rao JS, Shuber A, Markowitz SD (2005) Detection in fecal DNA of colon cancer-specific methylation of the nonexpressed vimentin gene. J Natl Cancer Inst 97: 1124–1132 16077070 10.1093/jnci/dji204

[bib8] Christensen E (1987) Multivariate survival analysis using Cox's regression model. Hepatology 7: 1346–1358 3679094 10.1002/hep.1840070628

[bib9] Colucci-Guyon E, Portier MM, Dunia I, Paulin D, Pournin S, Babinet C (1994) Mice lacking vimentin develop and reproduce without an obvious phenotype. Cell 79: 679–694 7954832 10.1016/0092-8674(94)90553-3

[bib10] Corsini MM, Miller RC, Haddock MG, Donohue JH, Farnell MB, Nagorney DM, Jatoi A, McWilliams RR, Kim GP, Bhatia S, Iott MJ, Gunderson LL (2008) Adjuvant radiotherapy and chemotherapy for pancreatic carcinoma: the Mayo Clinic experience (1975–2005). J Clin Oncol 26: 3511–3516 18640932 10.1200/JCO.2007.15.8782

[bib11] Dellagi K, Vainchenker W, Vinci G, Paulin D, Brouet JC (1983) Alteration of vimentin intermediate filament expression during differentiation of human hemopoietic cells. EMBO J 2: 1509–1514 11892803 10.1002/j.1460-2075.1983.tb01615.x PMC555314

[bib12] Domagala W, Lasota J, Dukowicz A, Markiewski M, Striker G, Weber K, Osborn M (1990a) Vimentin expression appears to be associated with poor prognosis in node-negative ductal NOS breast carcinomas. Am J Pathol 137: 1299–1304 1701960 PMC1877729

[bib13] Domagala W, Yuml;niak L, Lasota J, Weber K, Osborn M (1990b) Vimentin is preferentially expressed in high-grade ductal and medullary, but not in lobular breast carcinomas. Am J Pathol 137: 1059–1064 2173410 PMC1877664

[bib14] Goggins M (2011) Markers of pancreatic cancer: working toward early detection. Clin Cancer Res (in press) 10.1158/1078-0432.CCR-10-3074 PMC3079322 21304000

[bib15] Heatley M, Maxwell P, Whiteside C, Toner P (1993) Vimentin expression in benign and malignant breast epithelium. J Clin Pathol 46: 441–445 7686566 10.1136/jcp.46.5.441 PMC501254

[bib16] Herman JM, Swartz MJ, Hsu CC, Winter J, Pawlik TM, Sugar E, Robinson R, Laheru DA, Jaffee E, Hruban RH, Campbell KA, Wolfgang CL, Asrari F, Donehower R, Hidalgo M, Diaz Jr LA, Yeo C, Cameron JL, Schulick RD, Abrams R (2008) Analysis of fluorouracil-based adjuvant chemotherapy and radiation after pancreaticoduodenectomy for ductal adenocarcinoma of the pancreas: results of a large, prospectively collected database at the Johns Hopkins Hospital. J Clin Oncol 26: 3503–3510 18640931 10.1200/JCO.2007.15.8469 PMC3558690

[bib17] Hong SH, Misek DE, Wang H, Puravs E, Hinderer R, Giordano TJ, Greenson JK, Brenner DE, Simeone DM, Logsdon CD, Hanash SM (2006) Identification of a specific vimentin isoform that induces an antibody response in pancreatic cancer. Biomark Insights 1: 175–183 18769604 PMC2528299

[bib18] Hong SM, Li A, Olino K, Wolfgang CL, Herman JM, Schulick RD, Iacobuzio-Donahue C, Hruban RH, Goggins M (2011) Loss of E-cadherin xpression and outcome among patients with resectable pancreatic adenocarcinomas. Mod Pathol (in press) 10.1038/modpathol.2011.74 PMC3155013 21552209

[bib19] Hosmer DW, Lemeshow S (2000). Applied Logistic Regression 2nd edn Wiley: New York

[bib20] Iacobuzio-Donahue CA, Fu B, Yachida S, Luo M, Abe H, Henderson CM, Vilardell F, Wang Z, Keller JW, Banerjee P, Herman JM, Cameron JL, Yeo CJ, Halushka MK, Eshleman JR, Raben M, Klein AP, Hruban RH, Hidalgo M, Laheru D (2009) DPC4 gene status of the primary carcinoma correlates with patterns of failure in patients with pancreatic cancer. J Clin Oncol 27: 1806–1813 19273710 10.1200/JCO.2008.17.7188 PMC2668706

[bib21] Infante JR, Matsubayashi H, Tonascia J, Tonascia J, Klein AP, Riall TA, Yeo C, Iacobuzio-Donahue C, Goggins M (2007) Perineoplastical fibroblast SPARC expression and patient outcome with resectable pancreatic adenocarcinoma. J Clin Oncol 25: 319–325 17235047 10.1200/JCO.2006.07.8824

[bib22] Ivaska J, Pallari HM, Nevo J, Eriksson JE (2007) Novel functions of vimentin in cell adhesion, migration, and signaling. Exp Cell Res 313: 2050–2062 17512929 10.1016/j.yexcr.2007.03.040

[bib23] Javle MM, Gibbs JF, Iwata KK, Pak Y, Rutledge P, Yu J, Black JD, Tan D, Khoury T (2007) Epithelial-mesenchymal transition (EMT) and activated extracellular signal-regulated kinase (p-Erk) in surgically resected pancreatic cancer. Ann Surg Oncol 14: 3527–3533 17879119 10.1245/s10434-007-9540-3

[bib24] Kloppel G, Hruban RH, Longnecker DS, Adler G, Kern SE, Partanen TJ (2000) Ductal adenocarcinoma of the pancreas. In: Hamilton S, Aaltonen L (eds). WHO Classification of Digestive Neoplastia pp 220–230. IARC Press: Lyon

[bib25] Klymkowsky MW, Savagner P (2009) Epithelial-mesenchymal transition: a cancer researcher's conceptual friend and foe. Am J Pathol 174: 1588–1593 19342369 10.2353/ajpath.2009.080545 PMC2671246

[bib26] Lahat G, Zhu QS, Huang KL, Wang S, Bolshakov S, Liu J, Torres K, Langley RR, Lazar AJ, Hung MC, Lev D (2010) Vimentin is a novel anti-cancer therapeutic target; insights from *in vitro* and *in vivo* mice xenograft studies. PLoS One 16: e10105 10.1371/journal.pone.0010105 PMC2855704 20419128

[bib27] Lazarides E, Granger BL, Gard DL, O’Connor CM, Breckler J, Price M, Danto SI (1982) Desmin- and vimentin-containing filaments and their role in the assembly of the Z disk in muscle cells. Cold Spring Harb Symp Quant Biol 1: 351–378 10.1101/sqb.1982.046.01.036 7049530

[bib28] Leader M, Collins M, Patel J, Henry K (1987) Vimentin: an evaluation of its role as a tumour marker. Histopathology 11: 63–72 2435649 10.1111/j.1365-2559.1987.tb02609.x

[bib29] Li A, Omura N, Hong SM, Vincent A, Walter K, Griffith M, Borges M, Goggins M (2010) Pancreatic cancers epigenetically silence SIP1 and hypomethylate and overexpress miR-200a/200b in association with elevated circulating miR-200a and miR-200b levels. Cancer Res 1: 5226–5237 10.1158/0008-5472.CAN-09-4227 PMC3130565 20551052

[bib30] Liu LK, Jiang XY, Zhou XX, Wang DM, Song XL, Jiang HB (2010) Upregulation of vimentin and aberrant expression of E-cadherin/beta-catenin complex in oral squamous cell carcinomas: correlation with the clinicopathological features and patient outcome. Mod Pathol 23: 213–224 19915524 10.1038/modpathol.2009.160

[bib31] Matsubayashi H, Infante JR, Winter J, Klein AP, Schulick R, Hruban R, Visvanathan K, Goggins M (2007) Neoplastic COX-2 expression and prognosis of patients with resectable pancreatic cancer. Cancer Biol Ther 6: 1569–1575 18000398 10.4161/cbt.6.10.4711

[bib32] McInroy L, Määttä A (2007) Down-regulation of vimentin expression inhibits carcinoma cell migration and adhesion. Biochem Biophys Res Commun 17: 109–114 10.1016/j.bbrc.2007.06.036 17585878

[bib33] McShane LM, Altman DG, Sauerbrei W, Taube SE, Gion M, Clark GM, Statistics Subcommittee of the NCI-EORTC Working Group on Cancer Diagnostics (2005) Reporting recommendations for neoplastic marker prognostic studies. J Clin Oncol 23: 9067–9072 16172462 10.1200/JCO.2004.01.0454

[bib34] Medeiros LJ, Michie SA, Johnson DE, Warnke RA, Weiss LM (1988) An immunoperoxidase study of renal cell carcinomas: correlation with nuclear grade, cell type, and histologic pattern. Hum Pathol 19: 980–987 2456980 10.1016/s0046-8177(88)80016-9

[bib35] Naito Y, Iacobuzio-Donahue CA (2010) Biomarker profiles associated with metastatic pancreatic cancer. Modern Pathol 23: 366A

[bib36] Nakajima S, Doi R, Toyoda E, Tsuji S, Wada M, Koizumi M, Tulachan SS, Ito D, Kami K, Mori T, Kawaguchi Y, Fujimoto K, Hosotani R, Imamura M (2004) N-cadherin expression and epithelial-mesenchymal transition in pancreatic carcinoma. Clin Cancer Res 10: 4125–4133 15217949 10.1158/1078-0432.CCR-0578-03

[bib37] Neureiter D, Zopf S, Dimmler A, Stintzing S, Hahn EG, Kirchner T, Herold C, Ocker M (2005) Different capabilities of morphological pattern formation and its association with the expression of differentiation markers in a xenograft model of human pancreatic cancer cell lines. Pancreatology 5: 387–397 15980667 10.1159/000086539

[bib38] Nieminen M, Henttinen T, Merinen M, Marttila-Ichihara F, Eriksson JE, Jalkanen S (2006) Vimentin function in lymphocyte adhesion and transcellular migration. Nat Cell Biol 8: 156–162 16429129 10.1038/ncb1355

[bib39] Sato N, Fukushima N, Maehara N, Matsubayashi H, Koopmann J, Su GH, Hruban RH, Goggins M (2003) SPARC/osteonectin is a frequent target for aberrant methylation in pancreatic adenocarcinoma and a mediator of neoplastic-stromal interactions. Oncogene 22: 5021–5030 12902985 10.1038/sj.onc.1206807

[bib40] Schussler MH, Skoudy A, Ramaerkers F, Real FX (1992) Intermediate filaments as differentiation markers of normal pancreas and pancreas cancer. Am J Pathol 140: 559–568 1372155 PMC1886166

[bib41] Shirahata A, Sakata M, Sakuraba K, Goto T, Mizukami H, Saito M, Ishibashi K, Kigawa G, Nemoto H, Sanada Y, Hibi K (2009) Vimentin methylation as a marker for advanced colorectal carcinoma. Anticancer Res 29: 279–281 19331162

[bib42] Tascilar M, Skinner HG, Rosty C, Sohn T, Wilentz RE, Offerhaus GJ, Adsay V, Abrams RA, Cameron JL, Kern SE, Yeo CJ, Hruban RH, Goggins M (2001) The SMAD4 protein and prognosis of pancreatic ductal adenocarcinoma. Clin Cancer Res 7: 4115–4121 11751510

[bib43] Traub PG, Perides G, Scherbarth A, Traub U (1985) Tenacious binding of lipids to vimentin during its isolation and purification from Ehrlich ascites neoplastic cells. FEBS Lett 193: 217–221 4065338 10.1016/0014-5793(85)80155-1

[bib44] Vincent A, Herman JM, Schulick R, Hruban R, Goggins M (2011) Pancreatic cancer. Lancet (in press) 10.1016/S0140-6736(10)62307-0 PMC3062508 21620466

[bib45] Walter K, Omura N, Hong SM, Griffith M, Vincent A, Borges M, Goggins M (2010) Overexpression of smoothened activates the sonic hedgehog signaling pathway in pancreatic cancer-associated fibroblasts. Clin Cancer Res 16: 1781–1789 20215540 10.1158/1078-0432.CCR-09-1913 PMC2846609

[bib46] Winter JM, Cameron JL, Campbell KA, Arnold MA, Chang DC, Coleman J, Hodgin MB, Sauter PK, Hruban RH, Riall TS, Schulick RD, Choti MA, Lillemoe KD, Yeo CJ (2006) 1423 Pancreaticoduodenectomies for pancreatic cancer: a single-institution experience. J Gastrointest Surg 10: 1199–1210 17114007 10.1016/j.gassur.2006.08.018

[bib47] Winter JM, Ting AH, Vilardell F, Gallmeier E, Baylin SB, Hruban RH, Kern SE, Iacobuzio-Donahue CA (2008) Absence of E-cadherin expression distinguishes noncohesive from cohesive pancreatic cancer. Clin Cancer Res 14: 412–418 18223216 10.1158/1078-0432.CCR-07-0487 PMC3810144

[bib48] Zhao Y, Yan Q, Long X, Chen X, Wang Y (2008) Vimentin affects the mobility and invasiveness of prostate cancer cells. Cell Biochem Funct 26: 571–577 18464297 10.1002/cbf.1478

[bib49] Zhu QS, Rosenblatt K, Huang KL, Lahat G, Brobey R, Bolshakov S, Nguyen T, Ding Z, Belousov R, Bill K, Luo X, Lazar A, Dicker A, Mills GB, Hung MC, Lev D (2011) Vimentin is a novel AKT1 target mediating motility and invasion. Oncogene 30: 457–470 20856200 10.1038/onc.2010.421 PMC3010301

[bib50] Zou H, Harrington JJ, Shirc AM, Rego RL, Wang L, Campbell ME, Oberg AL, Ahlquist DA (2007) Highly methylated genes in colorectal neoplasia: implications for screening. Cancer Epidemiol Biomarkers Prev 16: 2686–2696 18086775 10.1158/1055-9965.EPI-07-0518

